# Pyorrhœa Not Infectious

**Published:** 1897-04

**Authors:** B. F. Arrington

**Affiliations:** GOLDSBORO, N. C.


ARTICLE VII.

### PYORRHOEA NOT INFECTIOUS.

BT DR. B. F. ARRINGTON, GOLDSBORO, N. C.

I have been very definitely convinced of one fact in relation to pyorrhœa. It is not contagious or transmissible from one person to another. I have watched carefully for results for more than a quarter of a century, and I am now thoroughly convinced that there is no such thing as transmitting from parent to child, or from any person to another under any circumstances. I have known several families residing in the country, embracing each parent and five or six children, where one or both parents were subject to the well defined, and all would use one co partnership toothbrush (a disgusting practice) until worn out, then another and so on, as needed, and not a child after a lapse of ten or fifteen years ever evinced the slightest feature of the disease. In one case in point, the husband, very loving and affectionate, fond of kissing his wife and children, was subject to the disease for more than thirty years (a typical case), losing by waste of process tooth after tooth, until but few remained in his mouth, and never did wife nor any of the children show signs of the disease. He (the husband) enjoyed good health, and he assured me more than once, he never suffered a moment from indigestion and experienced no particular discomfort from the disease.

I will mention one more case which I watched carefully for results for eighteen years, anticipating evil results from a censurable practice, but was disappointed. Mrs.—— died, leaving a boy child about six or eight months old, who was taken charge of and cared for by its grandfather and an old aunt, both subjects of pyorrhea well advanced, many teeth very loose, gums inflamed and swollen and blood and pus discharging under slightest pressure. The child was the pet of the family, and was tenderiy cared for, first one then the other, grandfather and aunt feeding it, and for months the process of feeding was as follows: One or the other would chew and pulp the food in the mouth then put it in the mouth of the child; so the food was prepared and administered until the child was able to masticate for itself. I advised against such a course and informed them of the chances and very great probability (as I then thought, do not now) of transmitting the disease to the chiid, but they heeded not. The child survived and grew up to manhood's years and stature, a splendid specimen of fine physique and blessed with as healthy gums and sound as could be desired. Should the young man be troubled with pyorrhœa (quite possible) after this, would any contend that the disease was transmitted through food pulped and administered by grandfather and aunt? I could state other cases almost as extreme and quite as convincing to unbiased minds that the disease is not transferable by any process of contact or through any channel of conveyance of disease.—*Southern Dental Journal.*

